# Newly developed TGF-β2 knock down transgenic mouse lines express TGF-β2 differently and its distribution in multiple tissues varies

**DOI:** 10.1186/1471-2091-14-21

**Published:** 2013-08-06

**Authors:** Yan-Bin XiYang, Fang Wang, Bao-Jiang Qian, Ling You, Bing-Tuan Lu, Wei Zhang, Xiong-Zhi Quan, Wen-Ping Ge, Su Liu, Lian-Feng Zhang, Ting-Hua Wang

**Affiliations:** 1 Institute of Neuroscience, Kunming Medical University, 1168 West Chunrong Road, Yuhua Avenue, Chenggong District, Kunming 650500, Yunnan, China; 2 Institute of Neurological Disease, Transformational Neuroscience Centre, West China Hospital, Sichuan University, NO. 17 Renmin South Road, Chengdu 610041, China; 3 Key Laboratory of Human Diseases Comparative Medicine, Ministry of Health, Chinese Academy of Medical Sciences (CAMS) & Comparative Medicine Centre, Peking Union Medical College (PUMC), NO. 5 Panjiayuan Nanli, Beijing 100021, China; 4 Institute of Laboratory Animal Science, Chinese Academy of Medical Sciences (CAMS) & Comparative Medicine Centre, Peking Union Medical College (PUMC), NO. 5 Panjiayuan Nanli, Beijing 100021, China

**Keywords:** TGF-β2, Knock down, Transgenic mouse, Protein levels, Distributions

## Abstract

**Background:**

Transforming growth factor-betas (TGF-βs), including beta2 (TGF-β2), constitute a superfamily of multifunctional cytokines with important implications in morphogenesis, cell differentiation and tissue remodeling. TGF-β2 is thought to play important roles in multiple developmental processes and neuron survival. However, before we carried out these investigations, a TGF-β2 gene down-regulated transgenic animal model was needed. In the present study, expressional silencing TGF-β2 was achieved by select predesigning interference short hairpin RNAs (shRNAs) targeting mouse TGF-β2 genes.

**Results:**

Four homozygous transgenic offspring were generated by genetic manipulation and the protein expressions of TGF-β2 were detected in different tissues of these mice. The transgenic mice were designated as Founder 66, Founder 16, Founder 53 and Founder 41. The rates of TGF-β2 down-expression in different transgenic mice were evaluated. The present study showed that different TGF-β2 expressions were detected in multiple tissues and protein levels of TGF-β2 decreased at different rates relative to that of wild type mice. The expressions of TGF-β2 proteins in transgenic mice (Founder 66) reduced most by 52%.

**Conclusions:**

The present study generated transgenic mice with TGF-β2 down-regulated, which established mice model for systemic exploring the possible roles of TGF-β2 *in vivo* in different pathology conditions.

## Background

Spinal cord injury (SCI) is a common medical problem, which can trigger a cascade of events, including infiltration by macrophages, activation of resident glial cells, formation of cavities in the injury site, axonal demyelination, loss of both sensory and motor neuron function and neuronal damage and death [1,2].

While numerous therapeutic interventions had been attempted in the past, a lack of suitable growth substrates, an insufficient activation of neuron-intrinsic regenerative programs, and extracellular inhibitors of regeneration limit the efficacy for anatomical and functional recovery after spinal cord injury [3]. The bulk of evidence has shown that the administration of some exogenous growth factors is potentially able to effect functional repair or nerveregeneration in injured spinal cords [4-6].

A large number of different cytokines/growth factors are secreted into spinal wounds by blood cells, platelets and endogenous cells. One superfamily of cytokines includes transforming growth factor-βs (TGFβs) [7], of which three isoforms, TGF-β1, -β2, and -β3, have been isolated in mammals [8]. It has been generally accepted that functions of TGF-β family members may vary depending on cellular status and cell types. TGF-β isoforms have been implicated in a broad diversity of biological activities, including cell growth, cell death, cell differentiation, inflammation, and immunological reactions, by modifying the expression of specific sets of target genes [9-11]. TGF-β has been shown to be both pro- and anti- apoptotic, influenced by both context and location. Increases or decreases in the production of TGF-β have been linked to numerous disease states, including atherosclerosis and fibrotic disease of the kidney, optical nerve, liver and lung. TGF-β, especially TGF-β2, is the predominant cytokine that plays an important role in the development of fibrosis [12-15]. Reports demonstrated that the later induction of TGF-β2 at the point of SCI may indicate a role in the maintenance of the scar [16]. It therefore suggested TGF-β2 is possibly involved in neuroplasticity following SCI. However, newly developed TGF-β2 knock down transgenic mouse lines express TGF-β2 is still needed.

In the present study, we established transgenic (Tg) mice with TGF-β2 knock down by genetic manipulation. Polymers chain reaction (PCR) was performed to identify the genotypes of mice. Then, Western blot and immunohistochemistry (IHC) were employed to detect the protein expressional levels and distributions of TGF-β2 in multiple tissues of different genotypes Tg mice. These tissues were olfactory bulb, cortex, frontal lobe, basal forebrain, cerebellum, hypothalamus, medulla oblongata, spinal cord, trachea, lung, heart, liver, spleen, kidney, adrenal gland, intestines, skeletal muscles and epidermis. The rates of TGF-β2 down-regulation in multiple tissues of different genotypes were evaluated by relative intensity to the level of wild type (WT).

## Results

### Genotypes detection of TG

**Figure 1:**
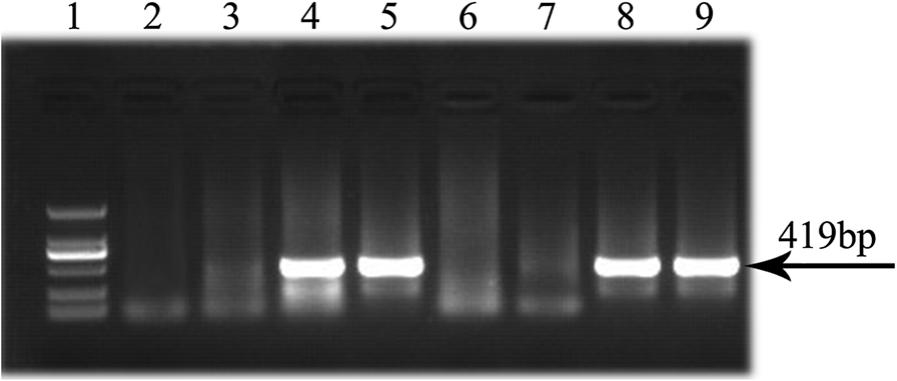
**Genotypes detection for the TGF-β2-kd Tg mice.** The positive Tg mice detected by PCR. Figure 1 showed the representative lanes of products electrophoresed in 1% agarose gel stained with EB. Lane 1: DNA Marker DL 2,000 (from up to down: 2000 bp, 1000 bp, 750 bp, 500 bp, 250 bp, 100 bp respectively). Lane 2–9: The PCR productions of inserted fragment from different heterozygous transgenic offspring of TGF-β2-kd lines. Lane 2, Lane 3, Lane 6 and Lane 7: WT; Lane 4: Founder 66; Lane 5: Founder 16; Lane 8: Founder 53; Lane 9: Founder 41.

Five heterozygosis transgenic offspring of TGF-β2-kd lines were obtained. Four of them could generate offspring, which were designated as Founder 66, Founder 16, Founder 53 and Founder 41. The Tg mice with inserted fragment, identified by PCR, were regarded as positive Tg (Figure 1).

### Protein expressional changes of TGF-β2 in multiple tissues of TG with different genotype

**Figure 2:**
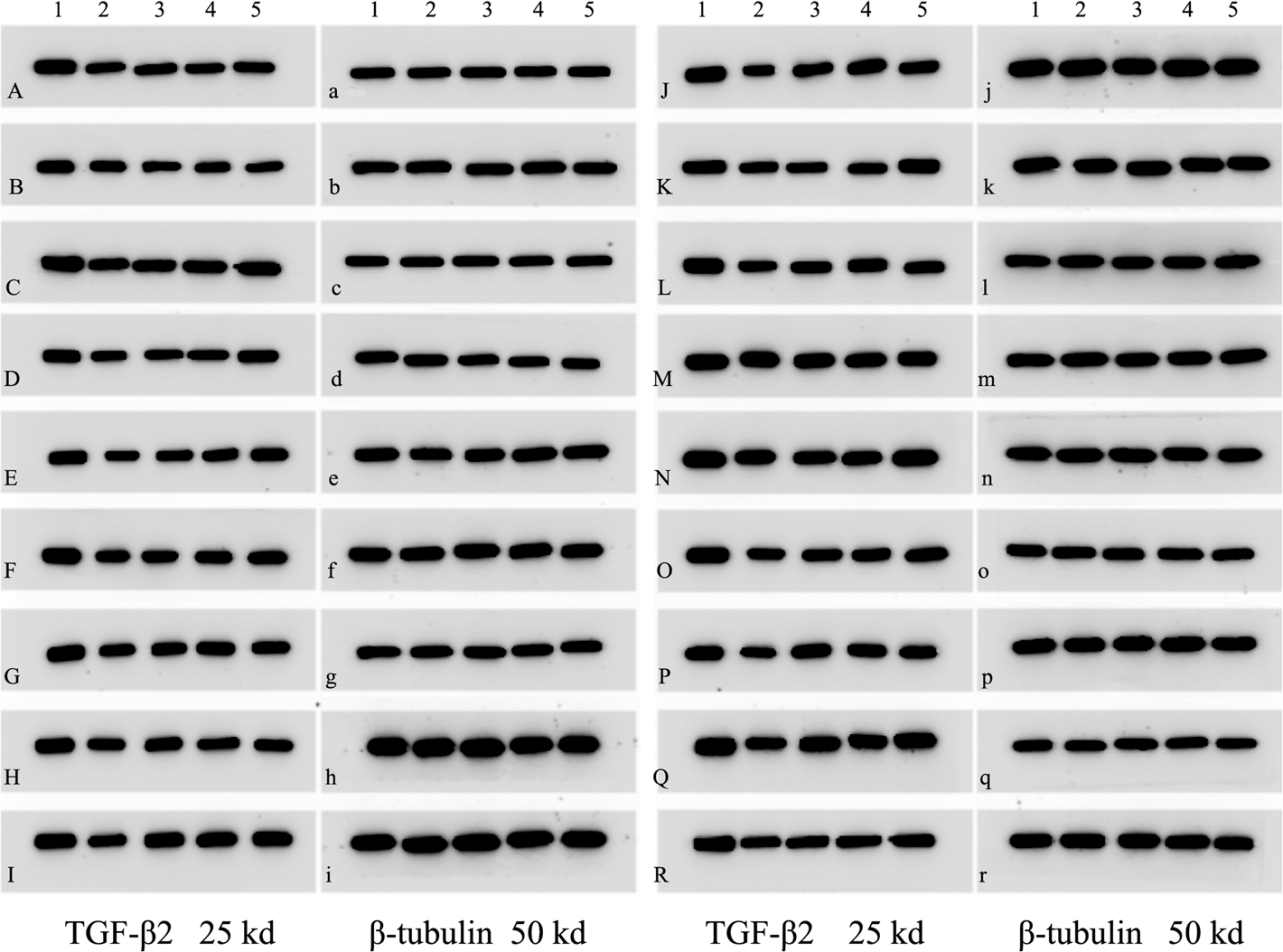
**Protein expressions of TGF-β2 detected by WB in different tissues.** Figure 2 Lane 1–5, TGF-β2 protein expression; Lane 1, WT; Lane 2, Founder 66; Lane 3, Founder 16; Lane 4, Founder 53; Lane 5, Founder 41. **A**, **a**: olfactory bulb; **B**, **b**: cortex; **C**, **c**: frontal lobe; **D**, **d**: basal forebrain; **E**, **e**: cerebellum; **F**, **f**: hypothalamus; **G**, **g**: medulla oblongata; **H**, **h**: spinal cord; **I**, **i**: trachea; **J**, **j**: lung; **K**, **k**: heart; **L**, **l**: liver; **M**, **m**: spleen; **N**, **n**: kidney; **O**, **o**: adrenal gland; **P**, **p**: intestines; **Q**, **q**: skeletal muscles; **R**, **r**: epidermis. Beta-tubulin was chased as the control.

**Figure 3:**
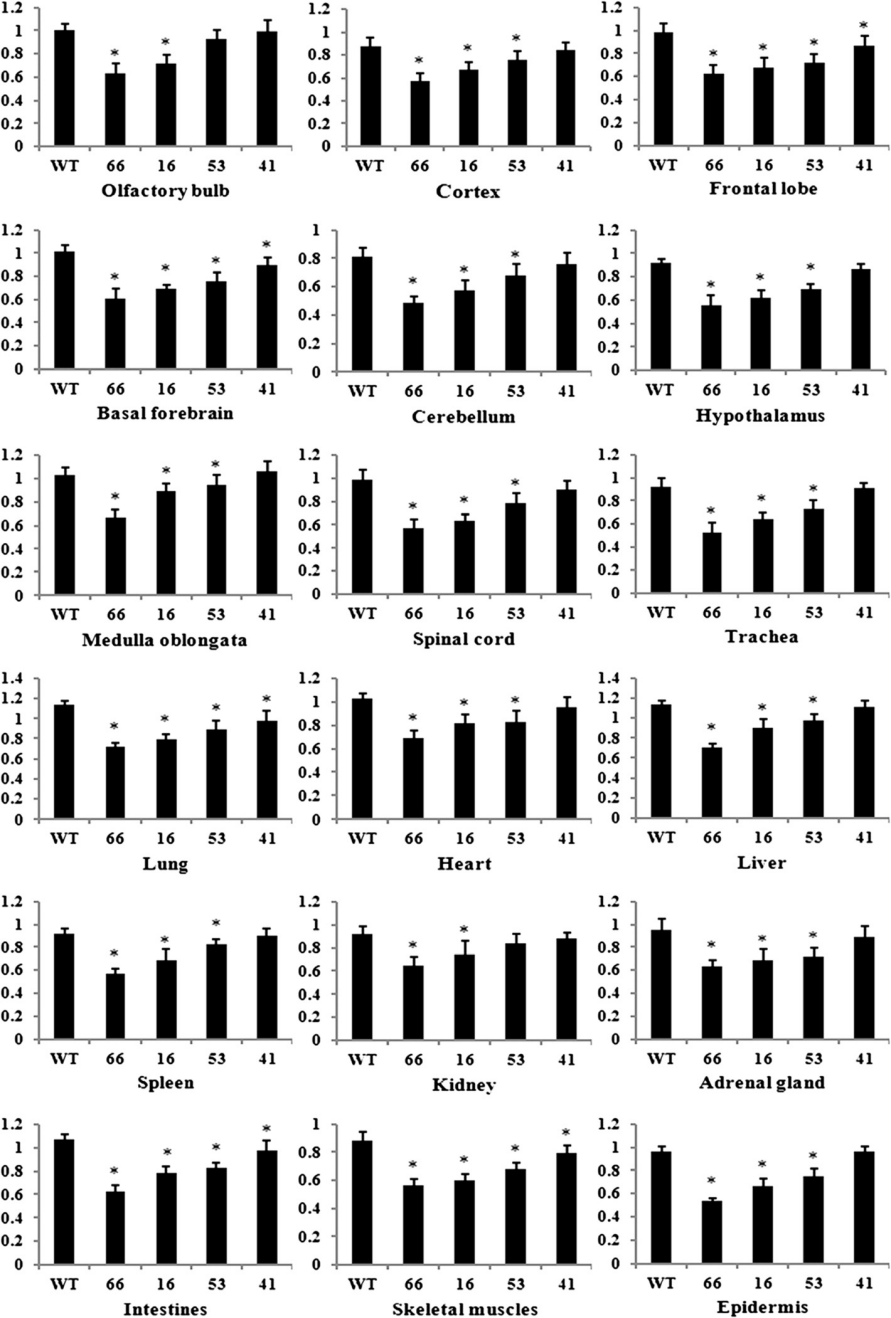
**Relative expressions of TGF-β2 in different tissues of Tg mice.** Figure 3 showed the relative optical density (O.D.) of TGF-β2 protein levels in multiple tissues of Tg mice and that of WT ones (n = 6). Values plotted are means ± SD. * compared with WT, *P <* 0.05. According to formula of the down-regulated rates of TGF-β2 protein, the average down-regulated rates of the four transgenic lines were calculated and described as followed. The average rates were 52%, 25%, 13% and 2% in Founder 66, Founder 16, Founder 53 and Founder 41, respectively. The relative expressions of TGF-β1 proteins in Founder 66, Founder 16 and Founder 53 were significantly different compared with that of WT mice (*P <* 0.05).

Results of Western blot, which detected in different multiple tissues of four genotypes TG (Founder 66, Founder 16, Founder 53 and Founder 41), indicated that TGF-β2 expressions were down-regulated by different percentages in the four kinds of TG mice (Figures 2 and 3). The rates of protein down-regulation were calculated as following: Rates of protein down-regulation = O.D. of WT- O.D. of Founder/O.D. of WT *100%. (O.D.: optical density).

### Distributions of TGF-β2 in multiple tissues

**Figure 4:**
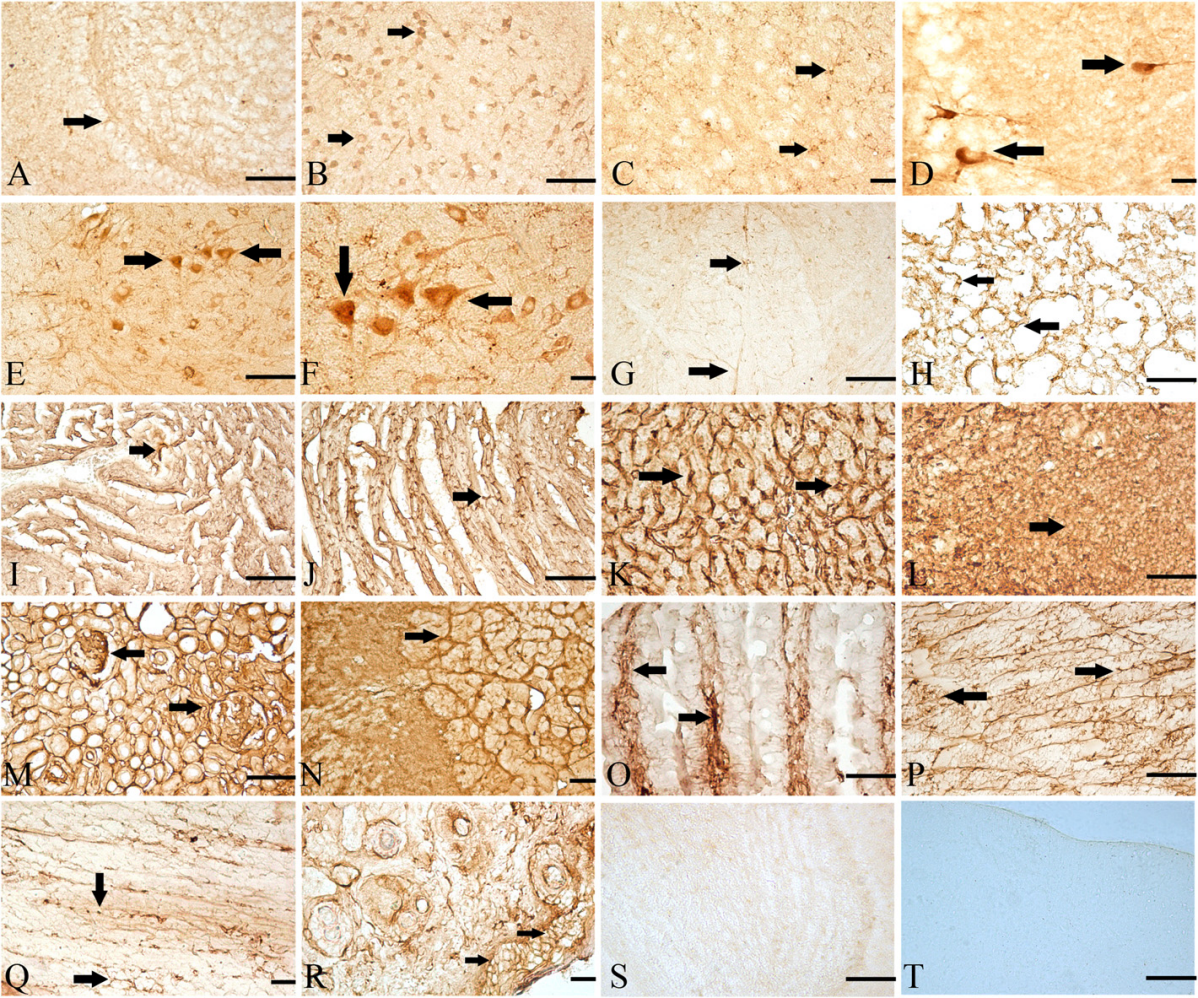
**Locations of TGF-β2 in multiply tissues of Tg mice.** The arrow showed the representative IR of TGF-β2 in Founder 66. **A**: olfactory bulb (arrow showed supporting cell); **B**: cortex frontal lobe (arrows showed neuron); **C**: basal forebrain (arrows showed neuron); **D**: hypothalamus (arrows showed neuron); **E**, **F** and **G**: spinal cord (arrows showed, **E** and **F**: motor neurons in the ventral horn); G: astrocyte-like cells in whiter matter); **H**: lung (arrows showed the lung epithelial cell); **I** and **J**: heart (arrows showed the sarcolemma); **K**: liver (arrows showed); **L**: spleen (arrow showed subendothelial smooth muscle cell); **M**: kidney (arrows showed epithelial cells); **N**: adrenal gland; **O**: intestines (arrows showed the staining lamina); **P** and **Q**: skeletal muscles (arrows showed the staining sarcolemma); **R**: epidermis (arrows showed the basal cells); **S**: control of olfactory bulb; **T**: control of brain. Magnifications: **C**, **D**, **N**, **Q** and **R**: 400×; other: 200×; Scale bar: 10 μm.

Control of immunostaining specificity was performed by replacing the primary antibody with 2% goat serum. These controls did not exhibit any specific immune-staining in the olfactory bulb and brain (Figure 4S and T, respectively).

#### Olfactory bulb

Immunoreactions (IR) of TGF-β2 was seen in basal cells, supporting cells, neurons, apical cytoplasmic region of olfactory epithelium, lamina propria and gland's cell cytoplasm. Positive-reactions were seen in a majority in the cytoplasm (Figure 4A).

#### Brain

The distributions of TGF-β2 immunopositive neurons and glia-liked cells were observed within the cortex, basal brain, frontal lobe, cerebellum, hypothalamus and medulla oblongata. They occurred in all layers of the cortical regions examined in this study, including the external and internal pyramidal layers. The somata and proximal dendrites with TGF-β2 IR were observed in the brain stem. A stronger labeling was present in granular cells and in axon-like fibers of the molecular cell layer. A few scattered immunopositive neuronal cell bodies and processes were present in the fastigial and dentate nucleus. Immunoreaction products of TGF-β2 were mainly observed in the cytoplasm and perikarya of these neurons. Nuclei of these cells were not stained (Figure 4B-D).

#### Spinal cord

TGF-β2 immunopositive profiles were present in rostral horn, ventral horn neurons as well as white matter of the spinal cord. The IR could be seen in the cytoplasm and processes, but not in the nucleus (Figure 4E-G).

#### Lung

TGF-β2 immunopositive profiles were found in the epithelial cells, vascular endothelial cells, as well as white blood cells. The IR was seen in the cytoplasm but not in the nuclei (Figure 4H).

#### Liver

TGF-β2 was distributed in the cytoplasm of hepatocytes throughout the liver lobule. The IR of TGF-β2 was partially seen in liver acinus (Figure 4K).

#### Spleen

IR of TGF-β2 was detected in Tunica media of artery, subendothelial smooth muscle cell and endotheliocyte. The immunoreactions then were seen in cytoplasm, but not in nucleus (Figure 4L).

#### Kidney

Representative IR for TGF-β2 in renal section of Tg mice showed diffuse positive staining within renal cortex, medullary interstitial, as well as the epithelial cells of the proximal convoluted tubule (Figure 4M).

#### Adrenal gland

The majority of TGF-β2 positive cells are located directly underneath the capsule, in the adrenal cortex (Figure 4N).

#### Intestine

TGF-β2 immunopositive files dispersed in lamina propria, epithelium mucosae and muscular layer. The immune-positive staining was primarily in the cytoplasm and partial cytolemma (Figure 4O).

#### Muscle

TGF-β2 staining was localized to the sarcolemma in skeletal muscle of mice. In the sarcoplasm there was staining in a transverse striation pattern at regular intervals the length of a sarcomere (Figure 4P and Q). Immunostaining for TGF-β2 also showed positive staining in coronary arteries of hearts (Figure 4I and J).

#### Epidermis

The positive-reactions of TGF-β2 were detected in the epidermis of TG mice. The IR was found in cytoplasm and cytolemma of basal cells and follicular epithelium (Figure 4R).

## Discussion

The present study generated different expression levels of TGF-β2 transgenic mice, which demonstrated that delivering shRNAs targeting TGF-β2 gene could induce TGF-β2 protein expression decrease in transgenic mice, especially in the central nervous system. Also, the expressed decrease in TGF-β2 protein was diverse in different phenotypic transgenic lines. The results detected by Western blot analysis showed that the lowest value (52%) of TGF-β2 protein was detected in Founder 66, while it was only 2% in Founder 41. In addition, we explored the systemic distribution of TGF-β2 in various tissues of TG mice, including the olfactory bulb, basal forebrain, cerebellum, cortex, hypothalamus, frontal lobe, medulla oblongata, spinal cord, lung, heart, liver, spleen, kidney, adrenal gland, intestines, skeletal muscles and epidermis. Newly developed Tg mice models of TGF-β2 down-regulation could be useful to further investigations.

Our results of PCR for genotypes detection, which showed that the inserted fragments (419 bp) were detected in four Tg offspring of TGF-β2-kd lines, indicated that new Tg mice model of TGF-β2-kd lines were obtained successfully by genetic manipulation. This study generated four kinds of available Tg mice, which were designated Founder 66, Founder 16, Founder 53 and Founder 41. These data strongly suggest that silence shRNAs for TGF-β2 can be used for the creation of a continuous mammalian model in which selected target genes are stably suppressed and attenuated *in vivo*.

RNA interference (RNAi) is an extremely effective tool for studying gene function in almost all metazoan and eukaryotic model systems. RNAi in mice, through the expression of short hairpin RNAs (shRNAs), offers something not easily achieved with traditional genetic approaches-inducible and reversible gene silencing. Previous research undertook targeted disruption of the TGF-β2 gene to determine its essential role *in vivo*. They demonstrated that TGF-β2-null mice exhibited perinatal mortality and a wide range of developmental defects for a single gene disruption. These include cardiac, lung, craniofacial, limb, spinal column, eye, inner ear and urogenital defects [17]. The present results show that shRNAs-TGF-β2 can induce extensive TGF-β2 down-regulation in mice. A random integration of a transgenic fragment effectively reduced the systemic expressions of TGF-β2 in Tg mice. However, the expression of decreased TGF-β2 protein was varied in different phenotypic lines, such that the highest rates of TGF-β2 down-expression (52%) was detected in Founder 66, while that of Founder 41 was only decreased by 2%. The diverse expression of TGF-β2 protein in four kinds of Tg mice might have been due to the randomness of insertion sites of the recombination vectors in the target gene. Furthermore, some unknown mechanisms of post-transcription regulation in different tissues might induce the different levels of TGF-β2 expressions in multiple tissues. Epigenetic deregulation of the TGF-β2 gene pathway members is likely to be an early event in breast cancer formation, which was resulted from the epigenetic regulation (such as histone methylation and deacetylation rather than DNA methylation) of TGF-β2 in a gene pathway [18]. In normal adult animals, TGF-βs (1–3) are ubiquitously and abundantly expressed in neurons and glia cells in both CNS and PNS [19-23]. The three TGF-beta isoforms described in mammals (TGF-β2, TGF-β2 and TGF-β3) have prominent functions related to morphogenetic events, epithelial-mesenchymal interactions, and differentiation [24,25]. A number of studies have suggested that TGF-beta1, 2, and 3 have differential temporal effects during the wound-healing process, and are important for optimal wound healing in the first week after wounding; beyond 1 week, TGF-beta1, 2, and 3 play a critical role in hypertrophic scar formation [26]. Furthermore, knockout mice have revealed their importance in regulating inflammation and tissue repair [27,28].

However, there is no report about newly developed TGF-β2 knock down transgenic mouse lines and the systemic distributions of TGF-β2 in Tg mice. The surveys of TGF-β2 distributions in newly developed TGF-β2 knock down transgenic mouse lines provided some crucial information to investigate the role of TGF-β2 under physiological and pathological condition.

In summary, this study developed Tg mice lines with TGF-β2 down-regulation and the systemic morphologic information that can be used in further research. Our results showed that TGF-β2 proteins were widespread in multiple tissues, especially in nervous systems, intestines and epidermis. These results indicated that TGF-β2 might play multiple different biologic roles according to the different cell types. Moreover, the present results generated four genotypes TGF-β2 Tg mice of expressional down-regulated by different folds, which supplied multiple genotypes Tg mice sources for different research.

## Conclusion

Our study established new transgenic mice lines with extensive down-regulation of TGF-β2. We also supplied the down-regulated rates and systemic distributions of TGF-β2 protein in four phenotypic transgenic mice. The results showed that TGF-β2 knockdown mice like Founder 66 could be designated as the target lines for further research.

## Methods

### Animal generation

Animal use and care were in accordance with the animal care guidelines, which conformed to the Guide for the Care and Use of Laboratory Animals published by the US National Institutes of Health (NIH Publication No. 85–23, revised 1996).

**Figure 5:**
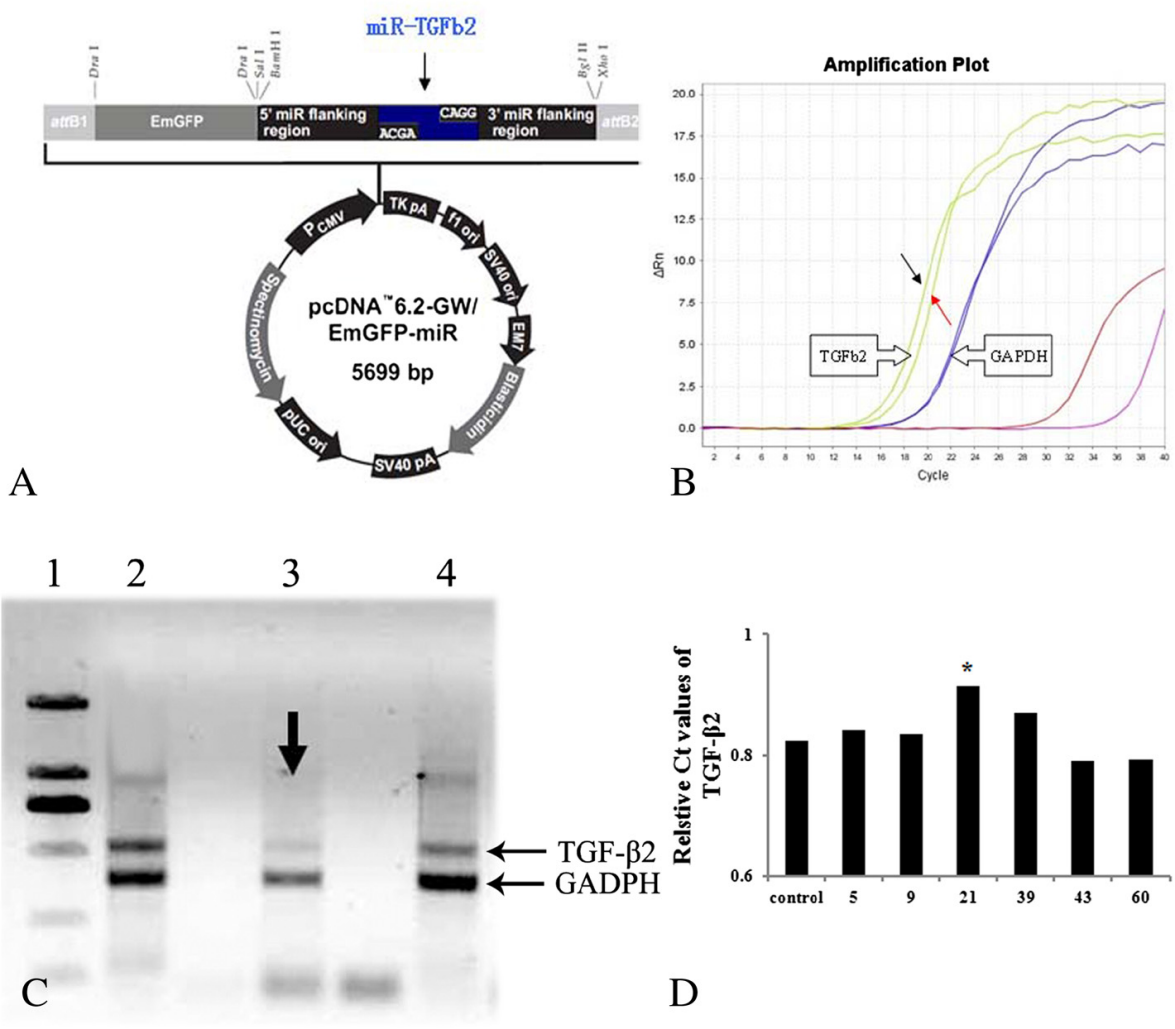
**Recombination plasmid for Tg mice with TGF-β2 down-regulation. A**: showed the schedules of recombination plasmid for pcDNA6.2-GW/EmGFP-miR of TGF-β2 gene silence, which composed with 5699 nucleotides. The 293T cells were transfected with the transgenic vectors (pcDNA3.1 (+) of pcDNA6.2-GW/EmGFP-miR-TGF-β1). RT-PCR was employed to evaluate the effects of PDGF-BB down-regulation transformants. **B**: showed the amplification plot of RT-PCR. Red arrows showed the selected cell lines as they had the lowest levels. Black arrow indicated the control ones. **C**: shows the represented bands of semi-quantity PCR products electrophoresed in 1% agarose gel stained with EB. Lane 1: DL2000 DNA Marker (from up to down: 2000 bp, 1000 bp, 750 bp, 500 bp, 250 bp, 100 bp respectively); Lane 2–4: 293T cells transfected with silence expression vector for TGF-β2 gene (lane 3: NO.21). Arrows in Figure 5C revealed the target transformants (NO.21) for TGF-β2 expressional silence as they had the lowest levels.

TGF-β2 knock down (TGF-β2-kd) transgenic (Tg) mice with C57BL/6J genetic background were produced by our collaborators in The Institute of Laboratory Animal Science (Chinese Academy of Medical Sciences & Comparative Medicine Centre, Peking Union Medical College, Beijing, China). The generation of the transgenic mice was described as follows. Briefly, at least three silence expression sites of TGF-β2 were designed by software supplied by Invitrogen Company, USA. Then we selected predesigned short hairpin RNA (shRNAs) that target mouse TGF-β2 gene (Mus musculus, GeneID: 21808). The reconstruction plasmid was designed (Figure 5A) and purchased from Invitrogen Company. The constructed recombinant plasmid was transferred into 293T cells. The transformants were screened and identified by polymers chain reaction (PCR) detections and restriction analysis (Figure 5B, C and D).

The protocol of PCR is described as follows. The transgene was then isolated from the cloning plasmid and purified by Avr II digestion, followed by diluted to a final concentration of 5 ng/μL. The final transgenic fragment was microinjected into fertilized mouse eggs (F1 [C57BL/6 × CBA/J] × F1 [C57BL/6 × CBA/J]). Detection for the transgenic fragment is described as follows.

Transgenic mice were mated with nontransgenic partners to maintain heterozygozity of the transgene or with transgenic partners to generate homozygous transgenic offspring. In the latter case, transgenic male mice were test mated with two wild-type female mice, and the offspring (15–20 individuals) was analyzed by polymerase chain reaction (PCR). Male mice that produced exclusively transgenic offspring were considered homozygous for the transgene. TGF-β2-kd Tg mice and their age-matched, non-transgenic littermates (wild type mice) were used.

### Real-time polymers chain reaction (RT-PCR)

The effects of shRNA target to TGF-β2 gene were detected by RT-PCR in transferred 293T cells. Total RNA was isolated from the harvested cells by using Trizol reagent (Invitrogen). cDNA was synthesized by using Oligo (dT) 18 and MMLV reverse transcriptase (Promega, Madison, WI). Primers employed were synthesized by Takara (Takara, Japan) and are described as follows. For detections in expression of TGF-β2 mRNA, the following primers were used: sense, 5′ CGGAGCATGGAAGTCACAG 3′; anti-sense, 5′ ACCACAGCCAGGAAACCC 3′. For GAPDH detections, the following primers were used: sense, 5′ CAAGGTCATCCATGACAACTTTG 3′; anti-sense, 5′ GTCCACCACCCTGTTGCTGTAG 3′. The cDNA was 10-fold serially diluted to seven concentrations for the standard curve.

RT-PCR protocol was applied using an ABI 5700 instrument (Bio-Rad). Reactions were performed in a 20 μl volume with 0.25 μM primers, 5 mM MgCl_2_, nucleotides, Taq DNA polymerase, and buffers were included in the DNA Master SYBR Green I mix (Applied Biosystems). Specificity of amplification products was confirmed by melting curve analysis. PCR was performed by the denaturation step at 95°C for 3 minutes, followed by 35 cycles of 95°C for 10 seconds, 55°C for 10 seconds, and 72°C for 30 seconds. Fluorescent signals from PCR products were recorded at 85.5°C for 5 seconds. TGF-β2 mRNA levels were normalized as the ratio of the fluorescence intensity from TGF-β2 to that of GAPDH.

### Semi-quantity PCR

Semi-quantity PCR analysis for the TGF-β2 expressions in transformants was performed. Prepare for RNA samples were described as above. Then the total RNA was eluted in 20 μl RNase-free Water (Gibco Life Technologies, Rockville, MD). The RNA was kept on ice and their concentrations were measured by a Nanodrop spectrophotometer (ND-1000). An equal amount of RNA (4 μg) was used for each experiment. The following primers were used: sense, 5′ CGGAGCATGGAAGTCA- CAG 3′; anti-sense, 5′ ACCACAGCCAGGAAACCC 3′. The product length of PCR is 512 bp. For GAPDH detections, the following primers were used: sense, 5′ CAAGGTCATCCATGACAACTTTG 3′; anti-sense, 5′ GTCCACCACCCTGTTGC- TGTAG 3′. The product length of PCR is 457 bp. Gene primers were synthesized by TaKaRa Company.

Experiments were duplicated to verify the results. For RNA amplification, the first-strand cDNA was synthesized from 4 μg of total RNA, using Revert AidTM First Strand cDNA Synthesis Kit (Fermentas Company, U.S.A.). PCR was then carried out using the PCR Master Mix Kit (Fermentas Company, U.S.A.) for 35 cycles, consisting of denaturation at 94°C for 1 min, annealing for 1 min, and extension at 72°C for 1 min. Then PCR products were electrophoresed in 1% agarose gel stained with ethidium bromide and visualized, using an ultra violet gel imager (BIO-GEL, BIP-RAD). The image analysis was performed by SYN Gene Tool (LIVE Science, U.S.A.).

### Assessment of genotypes

The inserted fragment was identified by PCR. For TGF-β2-kd lines, the following primers were used: sense, 5′GAGCAAAGACCCCAACGAG 3′; antisense, 5′TTATGA- ACAAACGACCCAACAC 3′. The lengths of PCR product is 419 bp. Briefly, PCR was carried out using the PCR Master Mix Kit (Fermentas Company, U.S.A.) for 35 cycles, consisting of denaturation at 94°C for 30 seconds, annealing at 60°C for 30 seconds, and extension at 72°C for 30 seconds. Then RT-PCR products were electrophoresed in 1% agarose gel stained with ethidium bromide and visualized, using an ultra violet gel imager (BIO-GEL, BIP-RAD). The image analysis was performed by SYN Gene Tool (LIVE Science, U.S.A.).

### Expressions of TGF-β2 Protein in different TG mouse

To investigate the level of TGF-β2 protein, multiple tissues including the olfactory bulb, cortex, frontal lobe, basal forebrain, cerebellum, hypothalamus, medulla oblongata, spinal cord, trachea, lung, heart, liver, spleen, kidney, adrenal gland, intestines, skeletal muscles and epidermis were obtained from mice with different genic genotypes. After carefully rinsing in cooled PBS, the hippocampus from each was homogenized on ice in a Lysis Buffer containing 0.05 M Tris–HCl (pH 7.4, Amresco), 0.5 M EDTA (Amresco), 30% TritonX-100 (Amresco), NaCl (Amresco), 10% SDS (Sigma) and 1 mM PMSF (Amresco), and centrifuged at 12,000rp for 30 min. The supernatant was then obtained and stored at −80°C for later use. Protein concentration was assayed with BCA reagent (Sigma, St. Louis, MO, USA). A 20 μl aliquot of the samples was loaded on to each lane and electrophoresed on 12% SDS-polyacrylamide gel (SDS-PAGE) for 2.5 h at a constant voltage of 120 V. Proteins were transferred from the gel to a nitrocellulose membrane for 6.5 h at 24 V. The membrane was blocked with phosphate-buffered saline containing 0.05% Tween-20 (PBST) with 10% nonfat dry milk overnight at 4°C for 12 h, then the membrane was washed three times for 10 min each time. They were then rinsed with PBST and incubated with the primary antibody for TGF-β2 (1:1000, Chemican) at 4°C for 24 h. After washing 3 times for 10 min each, the membrane was incubated with a HRP-conjugated goat anti-rabbit IgG (1:5,000; Vector Laboratories, CA) for 2 h at room temperature, and washing as described above. The membrane was developed in ECM kit, and then pictured by Bio-Gel Imagining system equipped with Genius synaptic gene tool software. Densitometry analysis for TGF-β2 protein was performed. β-tubulin (1:500, Santa cruz) was used as internal control.

### IHC

After anesthesia with 3.6% chloral hydrate (1 ml/100 g), mice were perfused with 150 ml of cold phosphate-buffered saline (PBS) for 5 min followed by 150 ml of 4% paraformaldehyde solution for 30 min. Multiple tissues described as above from each group was harvested, postfixed for 6-12 h, then immersed in 0.1 M PBS containing 20% sucrose overnight till the specimen sank to the bottom of the bottle. Sections of 20 μm thickness were cut in a freezing microtome (Leica CM1900, Germany), collected in a plate of 24 wells, rinsed with 0.01 M PBS three times, each for 5 min and soaked in PBS containing 3% H_2_O_2_ for 30 min at room temperature to block the endogenous peroxidase activity. After immersing in 0.01 M PBS containing 5% goat serum and 0.3% TritonX-100 solution at 37°C for 30 min, they were subsequently incubated at 4°C overnight with 2% goat serum containing goat polyclonal antibodies TGF-β2 (1:800, Santa Cruz). They were washed three times (5 min each time) in 0.01 M PBS containing 0.1% Tween-20 (PBST), and incubated in Reagents I and II from the PV-9000 Reagent Kit (Chemicon, Anti-Rabbit/Mouse Poly-HRP IHC Detection Kit, USA), each for 30 min at 37°C. It was again rinsed five times, each for 5 min in 0.01 M PBST. Finally, sections were detected by DAB staining. Negative control was performed by replacing the primary antibody with 2% goat serum to ascertain the specificity of antibody staining. IR products were observed and photographed with a light microscope (Leica. DMIRB, Germany) coupled with a computer assisted video camera.

## Competing interests

This work is supported by National Natural Science Foundation of China. NO. 81100911.

## Authors’ contributions

All authors have materially participated in the research and article preparation. Individual contribution to the article of each author is described as follow. YBX, LFZ and THW participated in the planning, execution, analysis of this study and manuscript preparation. FW, BJQ, LY and BTL were in charge of transgenic mice breeding and genotype detections. WZ, XZQ and WPG carried out animal generation. SL revised the manuscript. All authors read and approved the final manuscript.

## References

[B1] Fawcett JW Asher RA The glial scar and central nervous system repair Brain Res Bull 1999 49 377 391 10.1016/S0361-9230(99)00072-6 10483914

[B2] Beynon A Spinal cord injuries Nurs Stand 2011 25 59 21428263 10.7748/ns2011.03.25.26.59.c8372

[B3] McCall J Weidner N Blesch A Neurotrophic factors in combinatorial approaches for spinal cord regeneration Cell Tissue Res 2012 349 27 37 10.1007/s00441-012-1388-6 22526621 PMC3376183

[B4] Koda M Hashimoto M Murakami M Yoshinaga K Ikeda O Yamazaki M Adenovirus vector-mediated in vivo gene transfer of brain-derived neurotrophic factor (BDNF) promotes rubrospinal axonal regeneration and functional recovery after complete transection of the adult rat spinal cord J Neurotrauma 2004 21 329 337 10.1089/089771504322972112 15115607

[B5] Tang XQ Wang Y Huang ZH Han JS Wan Y Adenovirus-mediated delivery of GDNF ameliorates corticospinal neuronal atrophy and motor function deficits in rats with spinal cord injury Neuroreport 2004 15 425 429 10.1097/00001756-200403010-00009 15094497

[B6] Ruitenberg MJ Blits B Dijkhuizen PA te Beek ET Bakker A van Heerikhuize JJ Pool CW Hermens WT Boer GJ Verhaagen J Adeno-associated viral vector- mediated gene transfer of brain-derived neurotrophic factor reverses atrophy of rubrospinal neurons following both acute and chronic spinal cord injury Neurobiol Dis 2004 15 394 406 10.1016/j.nbd.2003.11.018 15006710

[B7] Noguti T Adachi-Yamada T Katagiri T Kawakami A Iwami M Ishibashi J Kataoka H Suzuki A Go M Ishizaki H Insect prothoracicotropic hormone: a new member of the vertebrate growth factor superfamily FEBS Lett 1995 376 251 256 10.1016/0014-5793(95)01296-8 7498553

[B8] Dobbertin A Schmid P Gelman M Glowinski J Mallat M Neurons promote macrophage proliferation by producing transforming growth factor-beta2 J Neurosci 1997 17 5305 5315 9204915 10.1523/JNEUROSCI.17-14-05305.1997 PMC6793830

[B9] Massague J TGF-beta signal transduction Annu Rev Biochem 1998 67 753 791 10.1146/annurev.biochem.67.1.753 9759503

[B10] Massague J Blain SW Lo RS TGFbeta signaling in growth control, cancer, and heritable disorders Cell 2000 103 295 309 10.1016/S0092-8674(00)00121-5 11057902

[B11] Anjaneyulu M Berent-Spillson A Inoue T Choi J Cherian K Russell JW Transforming growth factor-beta induces cellular injury in experimental diabetic neuropathy Exp Neurol 2008 211 469 479 10.1016/j.expneurol.2008.02.011 18406405 PMC2453508

[B12] Saika S TGF-beta signal transduction in corneal wound healing as a therapeutic target Cornea 2004 23 S25 S30 10.1097/01.ico.0000136668.41000.73 15448476

[B13] Zhang L Wang W Hayashi Y Jester JV Birk DE Gao M Liu CY Kao WW Karin M Xia Y A role for MEK kinase 1 in TGF-beta/activin-induced epithelium movement and embryonic eyelid closure EMBO J 2003 22 4443 4454 10.1093/emboj/cdg440 12941696 PMC202382

[B14] Krieglstein K Richter S Farkas L Schuster N Dünker N Oppenheim RW Unsicker K Reduction of endogenous transforming growth factors beta prevents ontogenetic neuron death Nat Neurosci 2000 3 1085 1090 10.1038/80598 11036264

[B15] Carrington LM Albon J Anderson I Kamma C Boulton M Differential regulation of key stages in early corneal wound healing by TGF-beta isoforms and their inhibitors Invest Ophthalmol Vis Sci 2006 47 1886 1894 10.1167/iovs.05-0635 16638995

[B16] Buss A Pech K Kakulas BA Martin D Schoenen J Noth J Brook GA TGF-beta1 and TGF-beta2 expression after traumatic human spinal cord injury Spinal Cord 2008 46 364 371 10.1038/sj.sc.3102148 18040277

[B17] Sanford LP Ormsby I Gittenberger-de Groot AC Sariola H Friedman R Boivin GP Cardell EL Doetschman T TGFbeta2 knockout mice have multiple developmental defects that are non-overlapping with other TGFbeta knockout phenotypes Development 1997 124 2659 2670 9217007 10.1242/dev.124.13.2659 PMC3850286

[B18] Hinshelwood RA Huschtscha LI Melki J Stirzaker C Abdipranoto A Vissel B Ravasi T Wells CA Hume DA Reddel RR Clark SJ Concordant epigenetic silencing of transforming growth factor-beta signaling pathway genes occurs early in breast carcinogenesis Cancer Res 2007 67 11517 11527 10.1158/0008-5472.CAN-07-1284 18089780

[B19] Flanders KC Ren RF Lippa CF Transforming growth factor-betas in neurodegenerative disease Prog Neurobiol 1998 54 71 85 10.1016/S0301-0082(97)00066-X 9460794

[B20] Lippa CF Smith TW Flanders KC Transforming growth factor-beta: neuronal and glial expression in CNS degenerative diseases Neurodegeneration 1995 4 425 432 10.1006/neur.1995.0051 8846236

[B21] De Groot CJ Montagne L Barten AD Sminia P Van Der Valk P Expression of transforming growth factor (TGF)-beta1, -beta2, and -beta3 isoforms and TGF-beta type I and type II receptors in multiple sclerosis lesions and human adult astrocyte cultures J Neuropathol Exp Neurol 1999 58 174 187 10.1097/00005072-199902000-00007 10029100

[B22] Lagord C Berry M Logan A Expression of TGFbeta2 but not TGFbeta1 correlates with the deposition of scar tissue in the lesioned spinal cord Mol Cell Neurosci 2002 20 69 92 10.1006/mcne.2002.1121 12056841

[B23] Peress NS Perillo E Differential expression of TGF-beta 1, 2 and 3 isotypes in Alzheimer's disease: a comparative immunohistochemical study with cerebral infarction, aged human and mouse control brains J Neuropathol Exp Neurol 1995 54 802 811 10.1097/00005072-199511000-00007 7595653

[B24] De Crescenzo G Grothe S Zwaagstra J Tsang M O'Connor-McCourt MD Real-time monitoring of the interactions of transforming growth factor- beta (TGF-beta) isoforms with latency-associated protein and the ectodomains of the TGF-beta type II and III receptors reveals different kinetic models and stoichiometries of binding J Biol Chem 2001 276 29632 29643 10.1074/jbc.M009765200 11382746

[B25] Goumans MJ Mummery C Functional analysis of the TGFbeta receptor/Smad pathway through gene ablation in mice Int J Dev Biol 2000 44 253 265 10853822

[B26] Lu L Saulis AS Liu WR Roy NK Chao JD Ledbetter S Mustoe TA The temporal effects of anti-TGF-beta1, 2, and 3 monoclonal antibody on wound healing and hypertrophic scar formation J Am Coll Surg 2005 201 391 397 10.1016/j.jamcollsurg.2005.03.032 16125072

[B27] Shull MM Ormsby I Kier AB Pawlowski S Diebold RJ Yin M Allen R Sidman C Proetzel G Calvin D Targeted disruption of the mouse transforming growth factor-beta 1 gene results in multifocal inflammatory disease Nature 1992 359 693 699 10.1038/359693a0 1436033 PMC3889166

[B28] Kulkarni AB Huh CG Becker D Geiser A Lyght M Flanders KC Roberts AB Sporn MB Ward JM Karlsson S Transforming growth factor beta 1 null mutation in mice causes excessive inflammatory response and early death Proc Natl Acad Sci USA 1993 90 770 774 10.1073/pnas.90.2.770 8421714 PMC45747

